# Suspension in Spinal Disease

**Published:** 1878-04

**Authors:** 


Suspension in Spinal Disease.—Dr. Bernard Roth (*Lancet*, Feb. 2d) says that the "so-called newly introduced method of suspension by the 'gallows' has been largely employed for many years past in several orthopædic institutions on the Continent;" that he saw last spring nearly the identical kind of apparatus used by Dr. Klopsch, of Breslau. "Here a strong horizontal beam is supported at its middle on a vertical mast fixed in the ground, and from the two extremities of the former depends exactly the same kind of gallows as is attached to the apex of Dr. Sayre's tripod. The patient's head having been fixed in the way now so well known here, the cord is shortened till the toes can only just touch the ground; the patient is now told to walk round and round, the horizontal beam being made to turn on a pivot. There are usually two or three cross-beams, and I have seen as many as six patients, walking round simultaneously as best they could on tip-toes, while their spines were being extended by the weight of their bodies. Complete suspension in the air is also employed very often. Even the shoulder-loops are quite identical with those now said to have been invented by the American professor. I saw a similar apparatus and treatment at the orthopædic institution of Dr. Schildbach, at Leipsic. This gentleman is a *privat docent* of, and has an orthopædic out-patients' department (*Policlinic*) in connection with the University of Leipsic. It is a curious fact that in Germany the gallows is called Glisson's swing (*Glisson'sche Schwebe*), after Francis Glisson, an English physician, who wrote a book, 'de Rhachetide,' a second edition of which appeared here in London in 1660. He quotes at page 368 a description of the swing, with an illustration, and explains the principle on which it is used. I have no wish to depreciate the great debt of gratitude we owe to Dr. Sayre for his introduction of the plaster-of-paris bandage for spinal disease, especially malum Pottii; but I do not think it is quite fair that no acknowledgment has been made of the real inventor of the suspender, nor of the fact of its having been so long in common use in Germany."—*Medical Record.*

